# Induced resistance in tomato by SAR activators during predisposing salinity stress

**DOI:** 10.3389/fpls.2013.00116

**Published:** 2013-05-06

**Authors:** Matthew F. Pye, Fumiaki Hakuno, James D. MacDonald, Richard M. Bostock

**Affiliations:** ^1^Department of Plant Pathology, University of California at Davis, DavisCA, USA; ^2^Research Center, Nihon Nohyaku Co., Ltd.Kawachi-Nagano,Osaka, Japan

**Keywords:** Tiadinil, Actigard, induced susceptibility, phytohormones, *Pseudomonas syringae* pv. *tomato*, *Phytophthora capsici*, predisposition

## Abstract

Plant activators are chemicals that induce disease resistance. The phytohormone salicylic acid (SA) is a crucial signal for systemic acquired resistance (SAR), and SA-mediated resistance is a target of several commercial plant activators, including Actigard (1,2,3-benzothiadiazole-7-thiocarboxylic acid-*S*-methyl-ester, BTH) and Tiadinil [*N*-(3-chloro-4-methylphenyl)-4-methyl-1,2,3-thiadiazole-5-carboxamide, TDL]. BTH and TDL were examined for their impact on abscisic acid (ABA)-mediated, salt-induced disease predisposition in tomato seedlings. A brief episode of salt stress to roots significantly increased the severity of disease caused by *Pseudomonas* *syringae* pv. *tomato* (*Pst*) and *Phytophthora capsici* relative to non-stressed plants. Root treatment with TDL induced resistance to *Pst* in leaves and provided protection in both non-stressed and salt-stressed seedlings in wild-type and highly susceptible NahG plants. Non-stressed and salt-stressed ABA-deficient *sitiens* mutants were highly resistant to *Pst*. Neither TDL nor BTH induced resistance to root infection by *Phytophthora capsici*, nor did they moderate the salt-induced increment in disease severity. Root treatment with these plant activators increased the levels of ABA in roots and shoots similar to levels observed in salt-stressed plants. The results indicate that SAR activators can protect tomato plants from bacterial speck disease under predisposing salt stress, and suggest that some SA-mediated defense responses function sufficiently in plants with elevated levels of ABA.

## INTRODUCTION

As sessile organisms, plants are presented with numerous biotic challenges such as herbivory and pathogen attack. Plants initiate responses to these challenges by harnessing tightly regulated phytohormone networks. Salicylic acid (SA) levels increase in plants following pathogen infection and SA is critical for the development of systemic acquired resistance (SAR; Métraux et al., 1990; Rasmussen et al., 1991). There are two enzymatic pathways for the generation of SA: one via phenylalanine ammonia lyase and the other via isochorismate synthase (ICS). In tomato (*Solanum lycopersicum*), *Arabidopsis* and *Nicotiana benthamiana*, most pathogen-induced SA appears to be synthesized via the ICS pathway (Wildermuth et al., 2001; Uppalapati et al., 2007; Catinot et al., 2008). Plants with compromised SA synthesis or signaling have greatly diminished defenses against pathogens, as is the case with SA-deficient transgenic plants expressing a bacterial salicylate hydroxylase (NahG; Gaffney et al., 1993) or ICS mutants like *sid2* (Wildermuth et al., 2001), and mutants in downstream targets of SA such as *npr1* (Mou et al., 2003). SAR induction by biotic agents coincides with increases in SA levels and a systemic transcriptional reprograming that primes the plant to respond rapidly to minimize the spread or severity of further infections (Malamy et al., 1990; Métraux et al., 1990; Rasmussen et al., 1991; Vlot et al., 2009). This transcriptional reprograming includes the expression of pathogenesis-related (PR) genes and deployment of peroxidases and other defense factors. In addition to induction by biotic agents, SAR responses are induced by exogenous application of SA to the foliage or roots (Ward et al., 1991).

Plant activators are chemicals that have no direct antimicrobial activity but induce disease resistance (Kessmann et al., 1994; Louws et al., 2001). A number of synthetic compounds have been developed that induce SAR by increasing SA accumulation (Iwai et al., 2007) and/or by acting on downstream targets of SA (Vernooij et al., 1995; Durrant and Dong, 2004). For example, the plant activator, probenazole, effective against bacterial, fungal, and oomycete diseases, stimulates SAR by increasing SA levels (Iwai et al., 2007). 1,2,3-Benzothiadiazole-7-thiocarboxylic acid-*S*-methyl-ester (BTH), sold under the trade name, Actigard, stimulates SAR in many plant species without inducing SA accumulation (Lawton et al., 1996). Tiadinil [TDL; *N*-(3-chloro-4-methylphenyl)-4-methyl-1,2,3-thiadiazole-5-carboxamide] is a plant activator that was registered in Japan in 2003 under the trade name, V-GET. TDL was developed for disease management in rice where it is applied to nursery-grown seedlings for transplanting to production fields (Tsubata et al., 2006). TDL is very effective for control of rice blast disease caused by *Magnaporthe oryzae* (Yasuda et al., 2006) and appears to induce resistance in a manner similar to BTH by acting on downstream targets of SA (Lawton et al., 1996; Yasuda et al., 2004). The TDL metabolite, 4-methyl-1,2,3-thiadiazole-5-carboxylic acid, is responsible for the SAR activation (Yasuda et al., 2006).

Abiotic stress alters the susceptibility of plants to many pathogens (Cho et al., 2009). The effect of brief episodes of root stress such as salinity and water deficit at levels that commonly occur in agriculture is well documented in plant–oomycete interactions, wherein stress events predispose plants to levels of inoculum they would normally resist (DiLeo et al., 2010). The phytohormone abscisic acid (ABA) accumulates rapidly in roots and shoots as an adaptive response to these abiotic stresses, but also contributes to the increased disease proneness of the plants (Mohr and Cahill, 2003; Thaler and Bostock, 2004; Fan et al., 2009; DiLeo et al., 2010). Antagonism between SA and ABA is well documented in relation to plant defense responses to pathogens (Mohr and Cahill, 2007; Jiang et al., 2010). Previously, ABA was found to have an antagonistic effect on SAR which was induced by 1,2-benzisothiazol-3(2*H*)-one1,1-dioxide and BTH in *Arabidopsis *and tobacco (Yasuda et al., 2008; Kusajima et al., 2010). However, it is not known if plant activators that target SA signaling impact the ABA-mediated susceptibility to root pathogens that occurs following predisposing root stress in tomato.

Because of the potential for unwanted tradeoffs and signaling conflicts in plants exposed to different stresses, as can occur in the field, we investigated how predisposing root stress impacts chemically induced resistance in tomato. The objective of this study was to determine the effect of pretreatment of tomato seedlings with TDL and BTH on salt-induced predisposition to the foliar bacterial pathogen *Pseudomonas syringae* pv.* tomato *(*Pst*) and to the soilborne oomycete pathogen* Phytophthora capsici*. TDL is of particular interest in the context of soilborne pathogens such as *Phytophthora capsici* because it is often applied to plants as a root dip. We also determined the impact of SA, TDL and BTH on ABA accumulation during a predisposing episode of salt stress. The results show that TDL applied to roots strongly protects the leaves from disease caused by *Pst* in both non-stressed and salt-stressed plants. In contrast, neither TDL nor BTH protects roots from *Phytophthora capsici*. The protection induced by plant activators against *Pst* does not result from reduced ABA accumulation and, although overall disease is less in both non-stressed and salt-stressed plants by chemically induced SAR, plant activators do not reverse the salt-induced increment in disease severity.

## MATERIALS AND METHODS

### PLANT MATERIAL AND GROWTH CONDITIONS

Tomato plants (*Solanum lycopersicum*) of cultivars “New Yorker” or “Rheinlands Ruhm” and mutants within these backgrounds were used in experiments. “New Yorker” seeds were obtained from a commercial source (Totally Tomatoes, Randolph, WI, USA). The homozygous ABA-deficient mutant *sitiens *was compared with its isogenic, wild-type (WT) background, “Rheinlands Ruhm” (Tal and Nevo, 1973), and seeds for these were obtained from the C.M. Rick Tomato Genetics Resource Center at the University of California, Davis. NahG transgenic plants were generated in the “New Yorker” background, similar to the method used by Gaffney et al. (1993). The *nahG* construct containing the transgene salicylate hydroxylase under control of the CaMV 35S promoter in the binary vector pCIB200 was a gift of Syngenta Crop Protection, Inc.

Tomato plants were grown in a hydroponic format. Prior to use, tomato seeds were surface sterilized with the following protocol: 50% HCl (10 min) and rinsed with sterile deionized H_2_O, 10% trisodium phosphate (15 min) and rinsed (3×) in sterile deionized H_2_O, 70% ethanol (10 min), and rinsed (3×) with sterile deionized H_2_O, and 50% commercial bleach (3% sodium hypochlorite; 20 min) followed by sterile deionized H_2_O rinse (3×). Following surface-sterilization, seeds were placed on sterile germination paper in beakers containing sterile deionized H_2_O, transferred after 1 week to trimmed 5 ml polypropylene pipette tips, secured with foam test tube plugs, and placed into aerated hydroponic containers filled with 4 L of aerated, 0.5× Hoagland’s solution. Seedlings were grown for an additional 2 weeks in a growth chamber (150 μmol m^–2^s^–1^, 16 h photoperiod, 22°C, 70% RH) until at least two true leaves had developed on each plant.

### SA TREATMENT, PLANT ACTIVATOR TREATMENT, SALT TREATMENTS, AND INOCULATION

Four-week-old hydroponically grown tomato plants were immersed in 50 ml of 0.5× Hoagland’s solution containing 10 ppm (37 μM) TDL (Nihon Nohyaku Co., Ltd), 10 ppm (47 μM) BTH (Syngenta Crop Protection, Inc.), 10 ppm (62 μM) salicylic acid-sodium salt (SA; Sigma-Aldrich), or water for 7 days prior to salt stress and inoculation with a pepper isolate of *Phytophthora capsici* (from Yolo County, CA; also pathogenic on tomato) or *Pst*, (isolate B-64, gift of D. Cooksey). Pre-inoculation salt treatments consisted of exposing the roots to saline solution (0.2 M NaCl + 0.02 M CaCl_2_) for 18 h. All seedlings collapsed within 10 min of exposure to saline solution and regained full turgor within 2 hr of salt removal. Shoots were dip inoculated with 2-day-old *Pst* cultures adjusted to 1 × 10^7^ cfu ml^-1^ in 1 L of 10 mM MgCl_2_ with 80 μl Silwet L77. Roots were inoculated with 2 ml of zoospore suspension to achieve a final concentration of 1 × 10^4^ zoospores ml^-1^.

### *Pst* AND *Phytophthora capsici* DISEASE ANALYSES

Four days post-inoculation (dpi) *Pst*-infected leaflets were surface sterilized with 70% EtOH for 10 s, rinsed in sterile H_2_O, and blotted dry. Samples were excised with a #3 hole punch (5 mm diameter) and ground in 200 μl 5 mM MgCl_2_. A series of 10-fold dilutions were plated on King’s B medium; colonies were counted after 2 days of growth at 28°C. The relationship of disease and *Phytophthora capsici* DNA content was determined by quantitative polymerase chain reaction (qPCR; DiLeo et al., 2010). To correct for variability across samples, a similar amount of hypocotyl and root tissue was extracted for each sample and the qPCR analyses were performed on DNA extracts adjusted for total DNA content as measured with a Nanodrop spectrophotometer model ND-1000 (Thermo Fisher Scientific Inc., Wilmington, DE, USA).

### ABA ANALYSES

To determine the effect of SA on ABA accumulation during salt stress, ABA levels were measured in WT plants pre-treated with SA, TDL, or BTH. Following salt stress treatment for 18 h, roots and shoots were collected and immediately frozen in liquid N_2_. The tissues were lyophilized and placed at -20°C until extraction. The lyophilized tissue was ground in liquid N_2_ to a fine powder with a mortar and pestle, 50–100 mg samples were collected, and each sample transferred to a microfuge tube. Cold 80% methanol (1.2 ml) containing butylated hydroxytoluene at 10 μg ml^-1^ was added to each tube, which was then vortexed. The extracts were placed on ice and agitated occasionally for 30 min. The tubes were centrifuged for 5 min at 10,000 × *g*, and the supernatants collected. The pellet was extracted with 0.5 ml of 80% methanol and centrifuged to collect the supernatant. This step was repeated, all three supernatants were combined, and the methanol concentration of the extract adjusted to 70%. The extracts were applied to pre-wetted Sep-pak C18 columns (Waters, Inc., Milford, MA, USA) and eluted with 5 ml of 70% methanol. The eluate (~7.5 ml) containing ABA was concentrated to near dryness at 37°C under vacuum and the volume adjusted to 300 μl with deionized water. The samples were analyzed by competitive immunoassay with an ABA immunoassay kit according to the manufacturer’s directions (Agdia/Phytodetek, Elkhart, IN, USA). Results are expressed as nanomoles of (+)-ABA per gram dry weight of tissue. To determine the effect of the *nahG* transgene on ABA levels, roots and shoots from WT and NahG plants were processed using the same procedure as above.

### SA ANALYSES

To determine the effect of the *nahG* transgene on SA accumulation following infection, SA was quantified in WT “New Yorker” and NahG backgrounds in non-inoculated plants and plants 3 dpi with *Pst*. Extraction of SA was carried out as previously described (Engelberth et al., 2004). Deuterated SA (C/D/N Isotopes, Inc., Quebec, Canada) was used as an internal standard. Methyl ester derivatives were analyzed by GC-MS in electronic ionization mode. Mass spectral analysis was done in selective ion monitoring mode. Fragment ions were SA-ME 152 and SA-D4-ME 156. Quantification calibration curves were generated with known quantities of pure SA.

### STATISTICAL ANALYSES

*Pseudomonas* *syringae* pv. *tomato* disease assays in “New Yorker” and “Rheinlands Ruhm” backgrounds were performed three times, with three replicates for each treatment within each experiment. The *Phytophthora capsici* disease assay experiment was performed three times with five replicates for each treatment within each experiment. Experiments measuring ABA accumulation were performed five times. SA accumulation was measured in one experiment with three replicates for each treatment. Statistical analysis was performed on all data sets. Log transformation was used for data which pass the Shapiro–Wilk’s test for normal distribution. The Tukey–Kramer test, Dunnett’s test, Wilcoxon rank sums test or *T*-tests were used for means comparisons using JMP software (version 10.0; SAS Inc.) as indicated.

## RESULTS

### TDL PROTECTS TOMATO AGAINST THE BACTERIAL SPECK PATHOGEN *Pst* IN NON-STRESSED AND SALT-STRESSED SEEDLINGS

**FIGURE 1:**
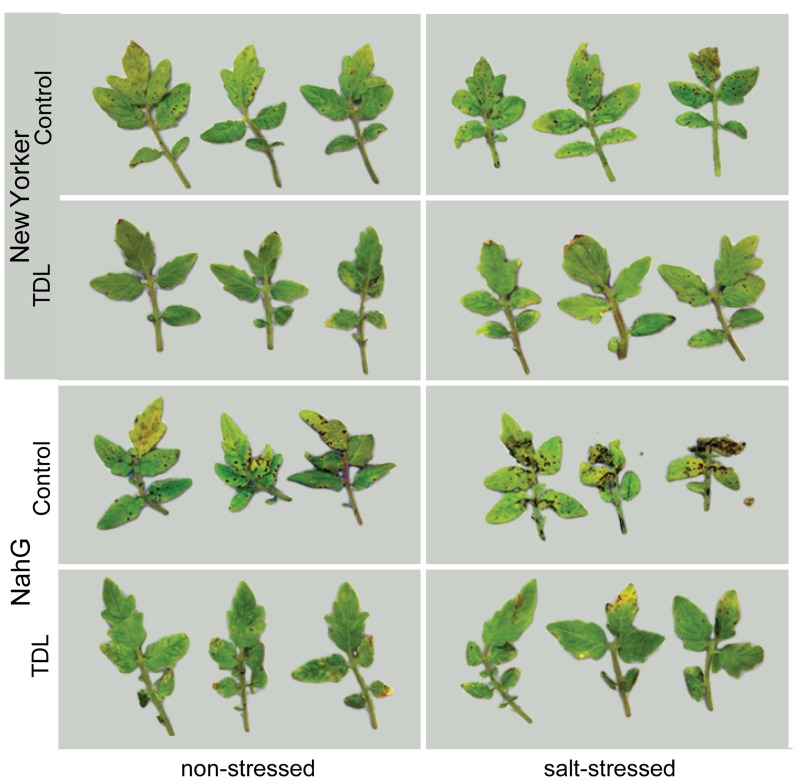
***Pseudomonas syringae* pv. *tomato* colonization inWT background (“New Yorker”) and NahG tomato leaves.** Roots were pretreated with TDL for 7 days and then exposed for 18 h to salt stress (0.2 M NaCl/0.02 M CaCl_2_). Shoots were dip-inoculated with a suspension of *Pst *adjusted to 10^7^ cfu ml^-1^. Symptoms photographed 4 dpi.

**FIGURE 2:**
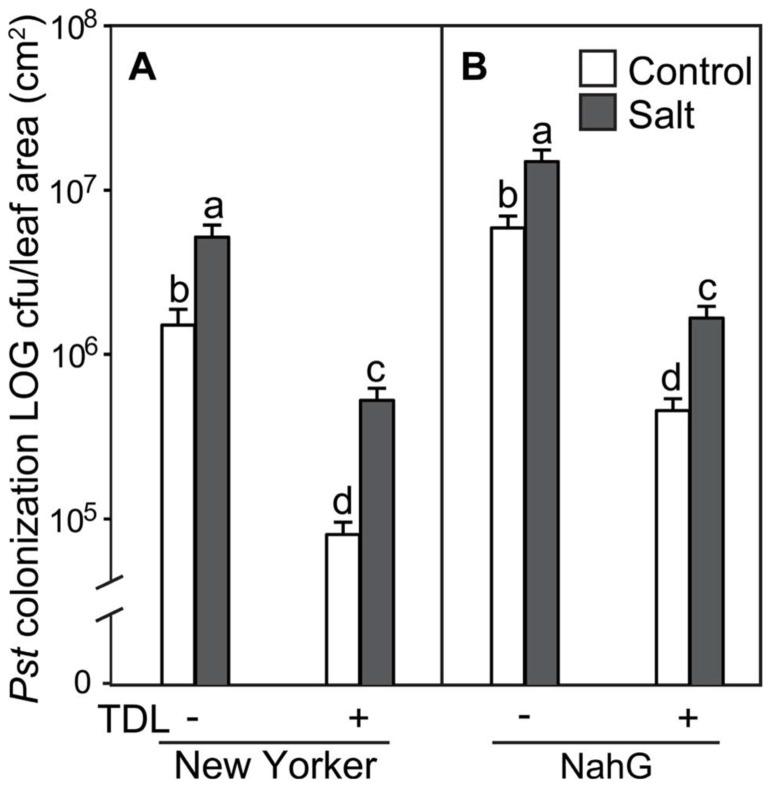
***Pseudomonas syringae* pv. *tomato* colonization in (A) WT background (“New Yorker”) and **(B)** NahG tomato leaves.** Roots were pretreated with TDL for 7 days and then exposed for 18 h to salt stress (0.2 M NaCl/0.02 M CaCl_2_). Shoots were dip-inoculated with a suspension of *Pst* adjusted to 10^7^ cfu ml^–1^. Colonization was evaluated 4 dpi. Bars represent the means ± SE from three expriments, *n* = 15. Letters above bars indicate significant differences between treatments at α = 0.05 using the Tukey–Kramer test for mean separation.

To determine if plant activators induce resistance to *Pst* under different stress regimes in our experimental format, roots of hydroponically grown seedlings of cv. “New Yorker” were treated with TDL and then either not salt-stressed or exposed to 0.2 M NaCl for 18 h prior to inoculation. In preliminary experiments, several concentrations of TDL were evaluated for phytotoxicity and for efficacy against bacterial speck disease with 10 ppm (37 μM) TDL selected as this concentration provided an optimal response. Concentrations higher than 10 ppm of TDL caused a slight bronzing of the roots and depressed growth of the seedlings, suggesting a mild phytotoxicity of the chemical in our experimental format at these higher levels. Inoculated salt-stressed seedlings had more severe disease symptoms (**Figure 1**) and a significantly higher titer of pathogen (**Figure 2**) than non-stressed, inoculated plants. Pretreatment with TDL at 10 ppm significantly reduced *Pst* colonization and symptom severity in “New Yorker” plants in both non-stressed and salt-treated seedlings (**Figure 2**). However, TDL did not prevent the proportional increase in *Pst* colonization observed in salt-stressed plants relative to the non-stressed controls.

Since TDL harnesses SA-mediated defenses, we treated SA-deficient NahG plants to see if TDL induces resistance under the different stress regimes in this highly susceptible background. As expected, NahG plants were more susceptible to *Pst* (**Figure 2**) and accumulated significantly less SA following *Pst *infection (data not shown) than the WT background “New Yorker.” However, TDL provided strong protection in the NahG plants and mitigated the predisposing effect of salt-stress on bacterial speck disease.

### TDL PROTECTS AGAINST *Pst* IN BOTH ABA-NORMAL AND ABA-DEFICIENT TOMATO SEEDLINGS

**FIGURE 3:**
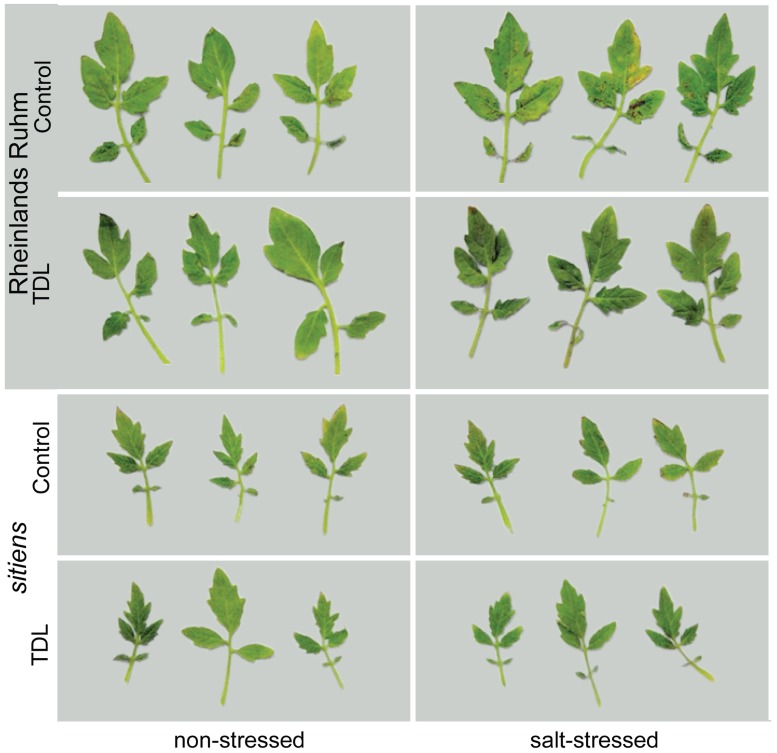
***Pseudomonassyringae* pv. *tomato* colonization in background (”Rheinlands Ruhm”) and *sitiens* tomato leaves.** Roots were pretreated with TDL for 7 days and then exposed for 18 h to salt stress (0.2 M NaCl/0.02 M CaCl_2_). Shoots were dip-inoculated with a suspension of *Pst *adjusted to 10^7^ cfu ml^-1^. Symptoms were photographed 4 dpi.

**FIGURE 4:**
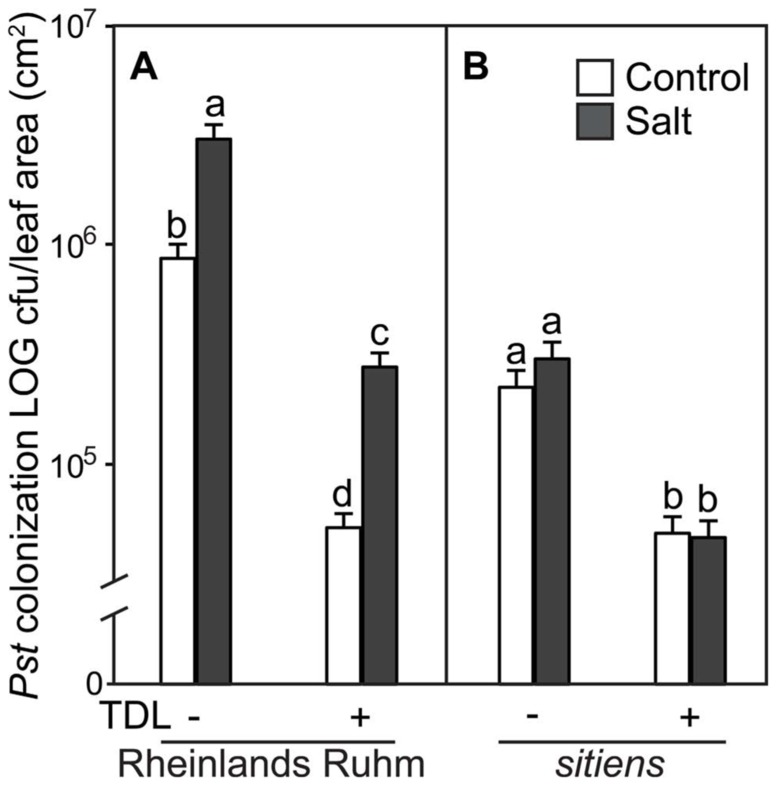
***Pseudomonassyringae* pv. *tomato* colonization in (A) WT background (“Rheinlands Ruhm”) and **(B)***sitiens* tomato leaves.** Roots were pretreated with TDL for 7 days and then exposed for 18 h to salt stress (0.2 M NaCl/0.02 M CaCl_2_). Shoots were dip-inoculated with a suspension of *Pst *adjusted to 10^7^ cfu ml^–1^. Colonization was evaluated 4 dpi. Bars represent the means ± SE from three expriments, *n* = 15. Letters above bars indicate significant differences between treatments at α = 0.05 using the Tukey–Kramer test for mean separation.

In a previous study we showed that ABA-deficient tomato mutants displayed a much reduced predisposition phenotype to salt stress (DiLeo et al., 2010). To determine if the protective effect of TDL is altered within an ABA-deficient tomato mutant, seedlings of WT (cv. “Rheinlands Ruhm”) and an ABA-deficient mutant within this background, *sitiens*, were treated in the same format and stress regimes as above. TDL significantly reduced *Pst* symptoms (**Figure 3**) and colonization (**Figure 4**) in both non-stressed and salt-treated plants of “Rheinlands Ruhm.” However, 3.6- and 5.4-fold increases in pathogen titer as a result of salt-stress were observed in both the control and TDL-treated plants, respectively, indicating that TDL did not prevent the proportional increase in *Pst* colonization in salt-stressed plants, similar to the results with “New Yorker” and NahG plants. In contrast, the *sitiens *mutant was not predisposed to *Pst* by salt stress and had significantly reduced symptoms (**Figure 3**) and colonization by the pathogen than the background “Rheinlands Ruhm” (**Figure 4**). Nonetheless, TDL pretreatment of *sitiens* provided further protection against *Pst *(**Figure 4**).

### TDL AND BTH DO NOT REDUCE *Phytophthora capsici* DISEASE SEVERITY

**FIGURE 5:**
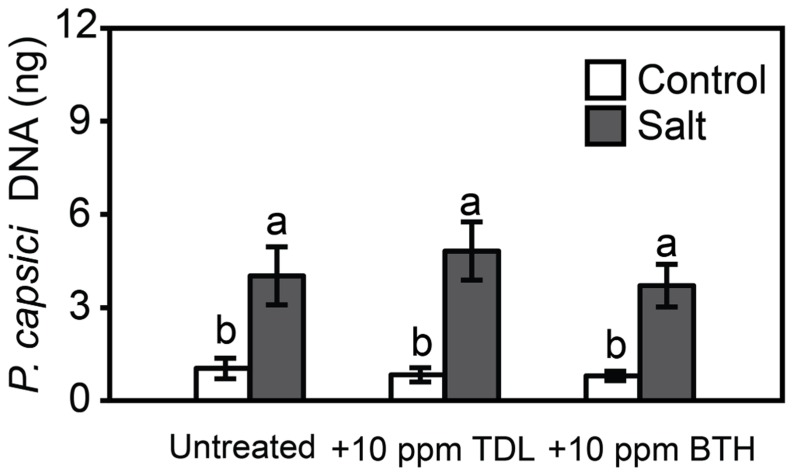
***Phytophthora capsici* colonization 48 hpi onWT “New Yorker” non-stressed (control) or salt-stressed (0.2 M NaCl/0.02 M CaCl_**2**_) roots for 18 h following pretreatment with TDL or BTH.** Colonization estimated by quantitative polymerase chain reaction of pathogen DNA. Bars represent the means ± SE from three experiments (*n* = 9 for each treatment). Letters indicate significant differences between treatments by *T*-test (α = 0.05).

To determine if plant activators protect tomato roots and crowns against the oomycete pathogen, *Phytophthora capsici*, and predisposing root stress, tomato seedlings were treated with TDL or BTH (10 ppm), not stressed or salt-stressed as above, and then inoculated. There was no protection provided by the plant activators against disease caused by *Phytophthora capsici* in either the control or salt-treated plants, as reflected in symptom severity (not shown) and pathogen colonization (**Figure 5**).

### IMPACT OF SALINITY STRESS AND PLANT ACTIVATORS ON ROOT AND SHOOT ABA LEVELS

**FIGURE 6:**
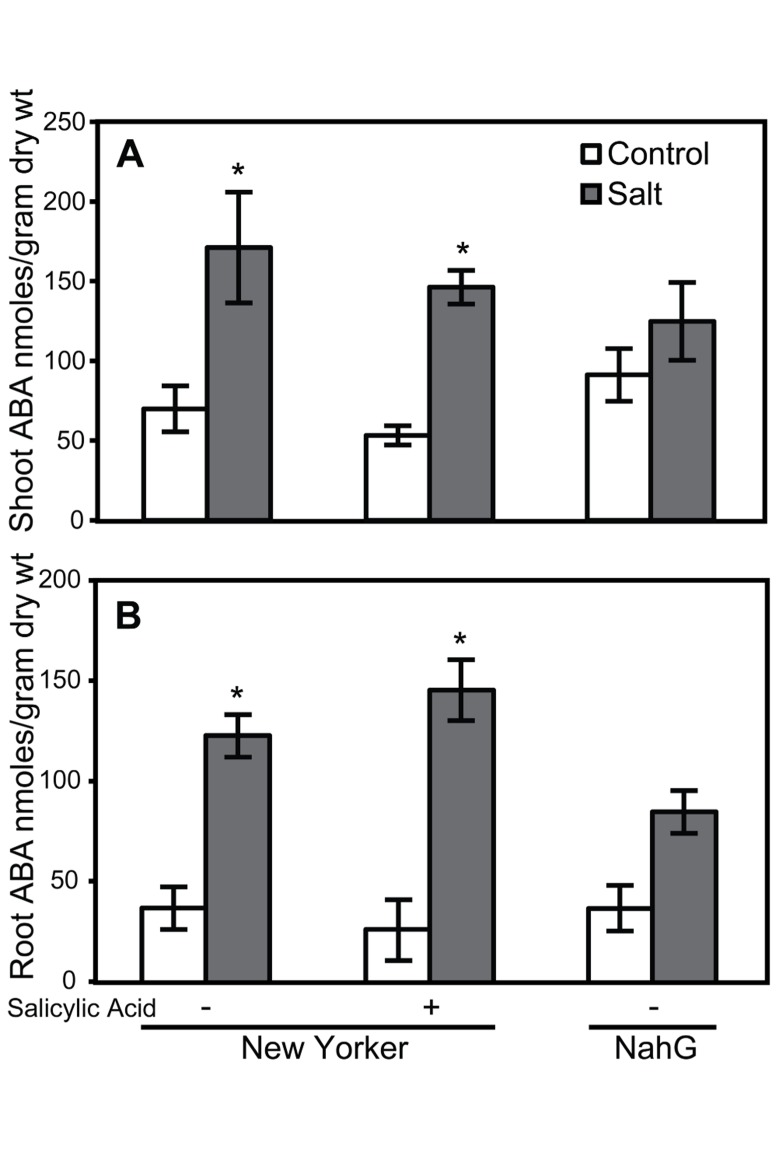
**ABA accumulation in shoots (A) and roots **(B)** of salt-stressed and non-stressed tomatoes with altered salicylic acid.** ABA levels in the roots of “New Yorker” and NahG seedlings, non-stressed (control) and 18 h salt-stressed (0.2 M NaCl and 0.02 CaCl_2_). + = seedling roots were treated with SA (62 μM) for 1 week prior to ABA measurement. Bars represent the means ± SE from five experiments (*n* = 15). Asterisks indicate significant differences over the “New Yorker” control by Dunnett’s test α = 0.05).

**FIGURE 7:**
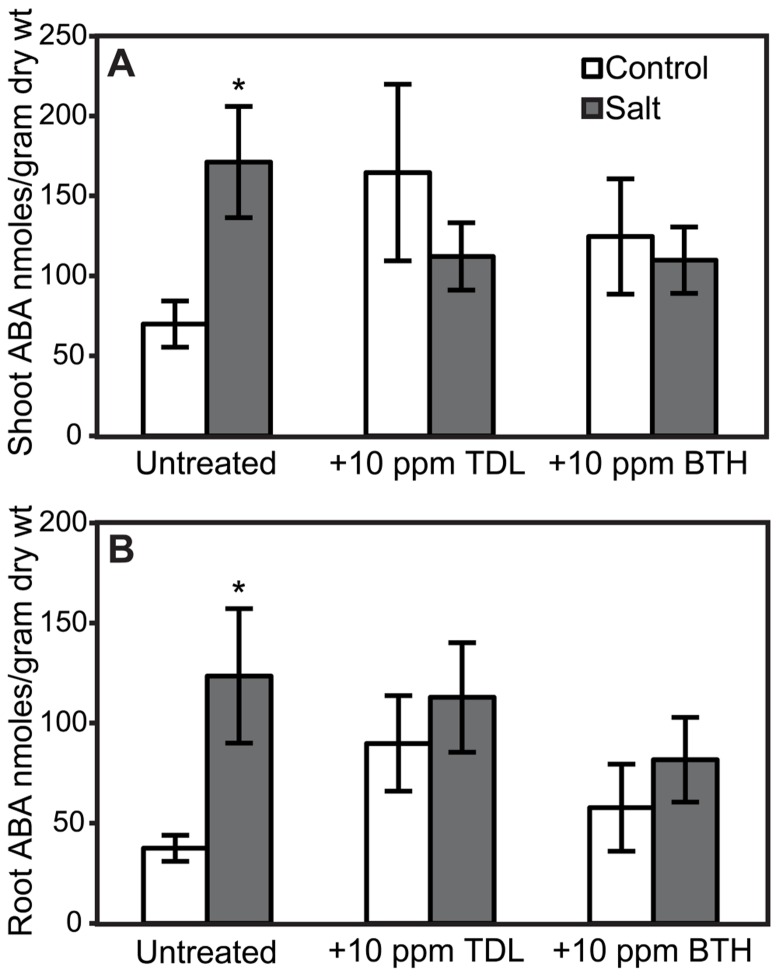
** ABA accumulation in shoots (A) and roots **(B)** of “New Yorker” plants non-stressed (control) or salt-stressed (0.2 M NaCl/0.02 M CaCl_**2**_) for 18 h, with and without prior TDL or BTH treatment.** Values are the means ± SE from three experiments (*n* = 9). Asterisks indicate a significant increase in shoot **(A)** (*χ*^2^ = 8.65, *P* = 0.003) and root **(B)** (*χ*^2^ = 5.78, *P* = 0.016) ABA in “New Yorker” salt over “New Yorker” control byWilcoxon rank sums.

Because elevated levels of ABA in tomato can enhance susceptibility to *Pst* (Mohr and Cahill, 2007) and *Phytophthora capsici *(DiLeo et al., 2010), the effect of SA, TDL, and BTH on ABA levels was determined in roots and shoots. ABA concentrations in either shoots or roots at the time selected for inoculation in our treatment sequence were not altered by SA (**Figure 6**). However, a trend of increasing ABA accumulation was observed in TDL- and BTH-treated “New Yorker” plants relative to the corresponding control plants (**Figure 7**). Although the increase in ABA accumulation in the plants treated with these plant activators is not statistically significant at *P* í 0.05, it can be said that SA, TDL, and BTH do not reduce ABA content relative to untreated plants (**Figure 7**). In addition, salt stress did not further increase the levels of ABA in plants that had been pretreated with TDL or BTH, which were similar to the salt stressed controls.

## DISCUSSION

In a previous study, we demonstrated the predisposing effect of salt stress and a role for ABA as a determinative factor in predisposition in the tomato–*Phytophthora capsici* interaction (DiLeo et al., 2010). The present study is the first report of salt-induced predisposition to the bacterial speck pathogen, *Pst*, in tomato. Furthermore, the results with the ABA-deficient *sitiens* mutant are consistent with the salt-induced susceptibility to *Pst* being mediated by ABA (**Figure 4**). These results conform to studies in *Arabidopsis* where ABA has been reported to promote susceptibility to *Pst* (de Torres-Zabala et al., 2007; Yasuda et al., 2008).

Because SA has been shown to protect tomato against salt stress, possibly by an ABA-dependent mechanism (Szepesi et al., 2009), plant activators that operate via the SA pathway were evaluated for effect on salt-induced predisposition. Protection of tomato against bacterial speck disease by BTH is well documented (Louws et al., 2001), and TDL has previously been shown to reduce the severity of bacterial and fungal infections without inducing SA accumulation (Yasuda et al., 2004, 2006). Here, TDL was shown to protect against *Pst *in both non-stressed and salt-stressed tomato plants. TDL pretreatment strongly reduced disease and colonization by *Pst *in both “New Yorker” and SA-deficient NahG plants. TDL, or more likely its biologically active metabolite, SV-03, presumably allows the NahG plants to mount an SAR response to *Pst* infection in the absence of SA accumulation (**Figure 2**). TDL provided protection in both non-stressed and salt-stressed plants, but did not reverse the predisposing effect of salt stress. An increase in *Pst* colonization was observed in the salt-stressed, TDL-pretreated plants of both genotypes, with comparable percentage increases relative to the corresponding non-stressed controls in “New Yorker” and NahG plants. This indicates that TDL does not reverse the salt-stress effect on disease, *per se*, and likely targets stress network signaling independently of an ABA-mediated process that conditions the salt-induced susceptibility observed in this system (**Figures 2** and **4**).

“Rheinlands Ruhm” also displayed salt-induced predisposition to *Pst.* Pretreatment with TDL significantly reduced *Pst* colonization in both “Rheinlands Ruhm” and *sitiens *(**Figure 4**). Similarly, TDL provided protection in both non-stressed and salt-stressed plants, but did not reverse the predisposing effect of salt stress in “Rheinlands Ruhm” plants. The salt-induced increment in colonization by the pathogen was comparable in both the untreated and TDL-treated plants (**Figure 4**). The ABA-deficient mutant, *sitiens*, is considerably less susceptible to *Pst* than its background “Rheinlands Ruhm,” and does not exhibit salt-induced predisposition (**Figures 3** and **4**).

Protection by plant activators against foliar pathogens is well established (Louws et al., 2001; Yasuda et al., 2004). However, relatively few studies have examined these compounds against soilborne pathogens and so TDL and BTH were evaluated for protection against root infection by *Phytophthora capsici*. Neither TDL nor BTH induced resistance or impacted salt-induced predisposition to *Phytophthora capsici* (**Figure 5**). *Phytophthora capsici* is an aggressive root and crown pathogen with a hemibiotrophic parasitic habit (Lamour et al., 2012) that triggers both SA- and jasmonic acid-mediated responses during infection of tomato (unpublished data). The results suggest that SA responses in tomato play a less important role in defense against *Phytophthora capsici *than to *Pst*.

The impact of SA and plant activators on ABA accumulation was measured in tomato roots and shoots. SA treatment and SA-deficiency conferred by NahG did not significantly impact ABA levels (**Figure 6**). However, ABA accumulation in non-stressed TDL and BTH treatments trended higher than those observed in salt-stressed plants that did not receive a plant activator treatment (**Figure 7**). Protection by TDL against *Pst* is likely the result of a triggered SAR response and not the result of an antagonistic effect on ABA levels.

The efficacy of plant activators depends on the specific diseases targeted and the environmental context, which may present additional stressors to confound defense network signaling in the plant. A challenge for successful deployment of plant activators in the field is to manage the allocation, ecological and fitness costs that are associated with induced defenses (Heil, 2001; Heil and Baldwin, 2002; Heil and Bostock, 2002; Berger et al., 2007). These costs can be manifested by reduced growth and reproduction, vulnerability to other forms of attack, and potential interference with beneficial associations (Bostock, 2005). It would seem that the severity of these costs is conditioned in part by the milieu of abiotic stressors operative at any given time. Reactive oxygen species (ROS) contribute to the initiation of SAR (Alvarez et al., 1998), are induced by SA and BTH (Fitzgerald et al., 2004; van der Merwe and Dubery, 2006), and are essential co-substrates for induced defense responses such as lignin synthesis (Hammerschmidt and Kuc, 1982). ROS also are important in modulating abiotic stress networks, for example in ABA signaling and response (Cho et al., 2009). The potential compounding effect of ROS generated from multiple stressors presents a dilemma in that the plant must reconcile these to adapt or else suffer the negative consequences of oxidative damage for failure to do so (Foyer and Noctor, 2009). Paradoxically, SA and BTH also are reported to protect plants against paraquat toxicity, which involves ROS generation for its herbicidal action (Silverman et al., 2005). How plants balance ROS’s signaling roles and destructive effects within multiple stress contexts is unresolved and a critically important area of plant biology with relevance for optimizing induced resistance strategies in crop protection (Van Breusegem et al., 2008; Foyer and Noctor, 2009). Although our experiments were conducted under highly controlled conditions, the results with TDL are encouraging and show that chemically induced resistance to bacterial speck disease occurs in both salt-stressed and non-stressed plants and in plants severely compromised in SA accumulation. Future research with plant activators should consider their use within different abiotic stress contexts to fully assess outcomes in disease and pest protection.

## Conflict of Interest Statement

This research was supported in part with funds from Nihon-Nohyaku Co., Ltd., the manufacturer of Tiadinil. These funds were unrestricted to support research and expenses associated with Fumiaki Hakuno’s sabbatical. We have no ongoing commercial or financial relationship with Nihon-Nohyaku Co., LTD. The purpose of this research was to provide an objective assessment of two commercial plant activators for their effects on disease within the context of predisposing abiotic stress.
